# Supplementary figures and images for: Decrease of heart rate variability during exercise: An index of cardiorespiratory fitness
Source: PLoS One. 2022 Sep 2;17(9):e0273981. doi: 10.1371/journal.pone.0273981 (PMC9439241; doi:10.1371/journal.pone.0273981)

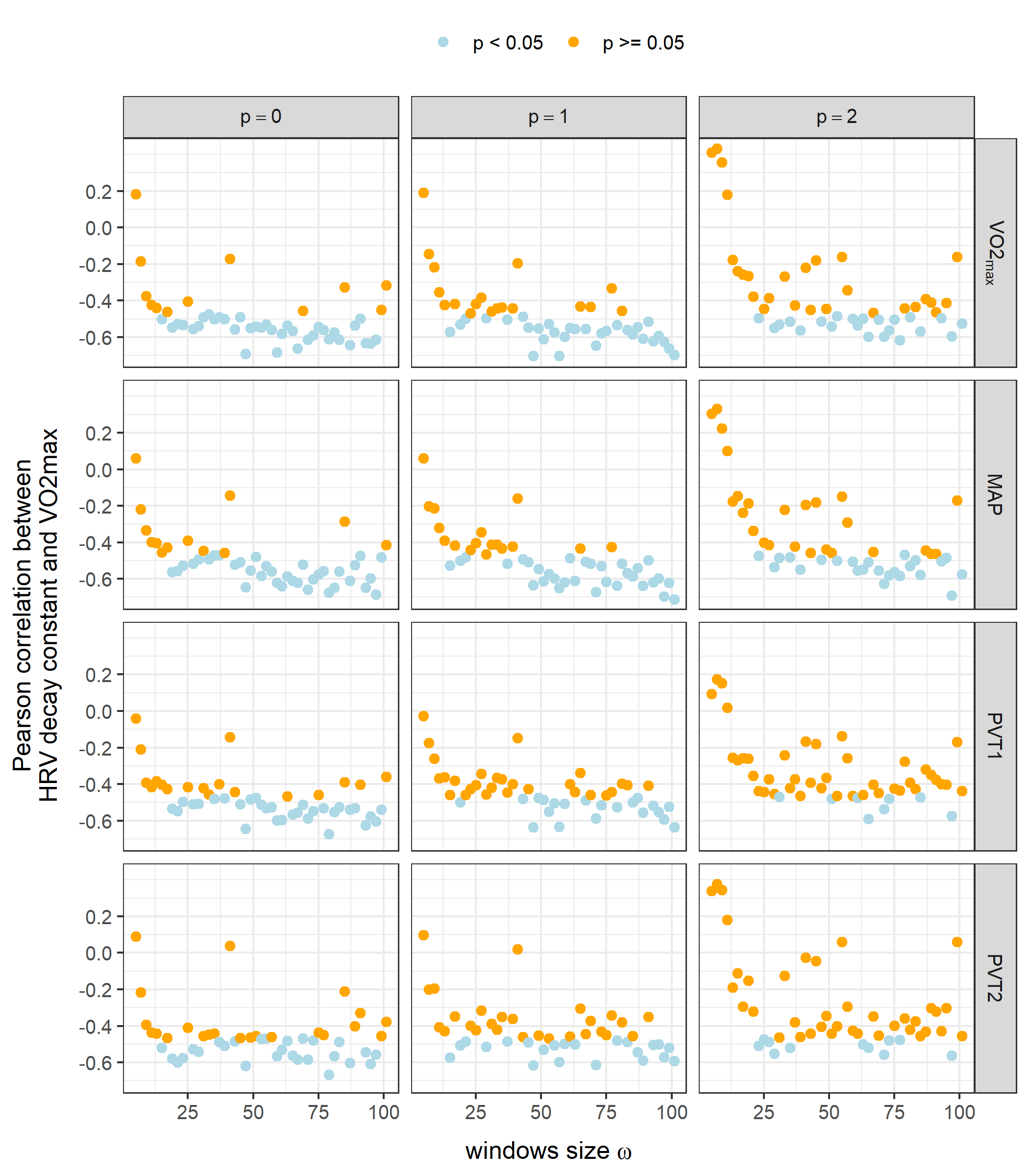

Supplement: S1 Fig — Pearson correlation coefficient and associated p value (p < 0.05 or p > = 0.05) found between HRV decay constant and VO2max, maximum aerobic power (MAP), power at the first (PVT1) and the second (PVT2) ventilator threshold as a function of the windows size ω when using a 0th (p = 0), first (p = 1) or second (p = 2) order polynomial detrending. (TIFF) [file pone.0273981.s001.tiff]
