# Supplementary material for: Decrease of heart rate variability during exercise: An index of cardiorespiratory fitness
Source: PLoS One. 2022 Sep 2;17(9):e0273981. doi: 10.1371/journal.pone.0273981 (PMC9439241; doi:10.1371/journal.pone.0273981)
Supplement: Attachment — Submitted filename: Revision Manuscript PONE.docx [file pone.0273981.s003.docx]

Revision Manuscript PONE-D-22-11838

This study investigates cardiac beat-by-beat measurements during maximal graded effort test; it can support other methods to consider the post-processing Heart Rate detections in promising tools for the study of cardiac measurements generated by portable devices with the aim of obtaining best performance in athletes. HRV was studied in function of the corresponding HR and it was confirmed that SDRR behaves as an exponential of the heart rate.

It seems very interesting, however, some points need to be better clarified. I think it is necessary, for example, in the introduction that a brief paragraph describing what CFR indices are and why it would be important to detect them.

The Akaike information criterion (AIC) and Bayesian Information criterion (BIC) can be briefly described in method or in appendix).

However, the main question is about the model that would describe the evolution of SDRR along the exercise (in the whole exercise duration time) by an exponential equation (in three different forms) in function of mean HR, power and workload. The parameter a and b in the equations are found by sliding windows along the exercise. Table 3 and Fig4 are refer only to model 1 (it may be better to change the title of the paragraph in “*Study of individual decrease of HRV during effort in model 1”),* this result must be emphasized.

The last part it is not clear in my opinion. How can Stationarity hypothesis in HR be assessed in window shorter than 30 sec (or 50 points)?

The fig.3 it is impressive, but it seems not to be necessary, or the significance must be better explained.

**Specific comments**

Abstract

Please rewrite the sentence: “It indicates that among athletes with better fitness, HRV has higher values at low heart rate and decreases faster when the heart rate increases during exercise”.

This is a key point. HRV has higher values at low heart rate it is well-known, it must be emphasized that HRV “decrease faster”

Introduction

How can measure the decrease of their variability in frequency domain?

Pag. 3

“such as the decrease of their variability measured both in time and frequency domain (8,10–15), the modification of the scaling properties of their linear correlations (16), or even the reduction of their sample entropy (17) and of their nonlinear correlations (18,19)”.

Too more information in two and half rows, please explain more in details and add even the more recent paper of [19] in PlosOne.

Pag 3 last row:

Add a definition and description cardiorespiratory fitness (CRF) indexes that are only mentioned at pag 4 :CRF indices (namely maximum VO2, maximum aerobic power, power at ventilator thresholds).

Pag 3 Row 15 replace work load with workload

Pag 3 i) Replace designed with defined

Please explain stationarity hypothesis in HR

Methods

Please, provide more information about sample.

For example: age, anthropometric measurements, explain why you can consider male and female together despite the well-known gender differences existing in physical exercises

calculated on adjacent windows of one minute (corresponding to each effort step during effort, see “Heart rate variability” subsection).

It is not clear and easy-readable: Please rewrite, describe the timing of protocol clearly, simply explain that the workload changes every minute. (see pag7)

Pag 7 “Detrending”: what are the differences among 23-24-25?

AIC and BIC can be briefly descried in method or in appendix

Pag 7 last rows

Because HRV is a result of parasympathetic and sympathetic neuronal activity, both being processes taking part in the regulation of the mean HR, we propose to analyze the decay of SDRR during exercise as a function of the mean HR calculated on the same time windows.

It is not clear because the second part (“we propose to analyze …” is a consequence of the first part “Because HRV is …)

Results

Pag 9 Explain better the first three rows

“The median number of RR distance recorded was 2726 beats (Inter Quartile Range [2433;3548]). An example of detrended RR estimated using the dynamical model presented in the method section is illustrated in Fig 2. This model produced an individual estimation of RR with a median R2 of 0.97 [IQR: 0.91 – 0.98].

Pag. 10 add to the first sentence something like: “for these reason in the following ……only model 1…”

In Fig3 Please add three panels with only “test”

In Caption of fig3 A comma is missing before “respectively”: in A, B and C, respectively

Pag 12 table please correct “correlation”

(HRV decay rate a and the HRV at HR=0 b) change “a, HRV decay rate and b, HRV decay rate at HR=0 “

HRV was studied in function of the corresponding HR:

Can you better explain the difference between ω_RR_ and ω_p_ ?

Please explain because it would be trivial to consider ω_RR_ and ω_p_  with the same number of points

In my opinion it is not clear to catch the differences between ω_RR_ and ω_p_

Pag 13 athletes with higher aerobic capacity….. Add references

In the fig3, to what correspond the horizontal line below 50 appears in all the panels?
