# Supplementary material for: Decrease of heart rate variability during exercise: An index of cardiorespiratory fitness
Source: PLoS One. 2022 Sep 2;17(9):e0273981. doi: 10.1371/journal.pone.0273981 (PMC9439241; doi:10.1371/journal.pone.0273981)
Supplement: Attachment — Submitted filename: reviewer_answer_V2.docx [file pone.0273981.s004.docx]

Dear Professor Laurent Mourot, dear reviewers

Please find our revised version of our article «Decrease of heart rate variability during exercise: an index of cardiorespiratory fitness» and the associated detailed answer to the reviewers remarks.

We would like to thank the editorial board of PlosOne and the reviewers for the quality of the review. The remarks were pertinent and accurate, and helped us improving our manuscript.

Our answer are in **bold**, and citation from the text are in ***italic***.
We made substantial changes to meet the reviewers’ expectations. Below are the main changes:

- We tried to clarify where our study stands compared to the work of Lewis and co-authors, who first developed the idea of the HRV decay constant. The main novelty of our approach is to compare several models and to make use of a cardiac measurements only based approach. We show that this approach allows to obtain results as good as those presented by Lewis and co-authors, but can technically be applied to cardiac measurements produced by portable devices.
- In order to ease the comparison with the above-mentioned work of Lewis and co-authors, we used the same definition of the HRV decay constant, instead of the HRV decay rate we used in the last version of our work
- In order to ease its understanding, we modified our sensitivity analysis so that the size of the time windows of the polynomial detrending and of the time windows of the SDRR calculation are the same parameter $\omega$.

The results, the figures, and the R code available in the Gitlab repository have been updated accordingly.
We hope this new version of our manuscript will meet the reviewers’ expectations and will be suitable for publication in PlosOne.

Denis Mongin, In behalf of the authors

# Journal Requirements:

When submitting your revision, we need you to address these additional requirements.1.

1. Please ensure that your manuscript meets PLOS ONE's style requirements, including those for file naming. The PLOS ONE style templates can be found at

<https://journals.plos.org/plosone/s/file?id=ba62/PLOSOne_formatting_sample_title_authors_affiliations.pdf>

**We modified the text so it meets PLOS ONE’s style requirements**

2. Please include a complete copy of PLOS’ questionnaire on inclusivity in global research in your revised manuscript. Our policy for research in this area aims to improve transparency in the reporting of research performed outside of researchers’ own country or community. The policy applies to researchers who have travelled to a different country to conduct research, research with Indigenous populations or their lands, and research on cultural artefacts. The questionnaire can also be requested at the journal’s discretion for any other submissions, even if these conditions are not met.  Please find more information on the policy and a link to download a blank copy of the questionnaire here: https://journals.plos.org/plosone/s/best-practices-in-research-reporting. Please upload a completed version of your questionnaire as Supporting Information when you resubmit your manuscript.

**We uploaded the questionnaire with the revised manuscript.**

3. Thank you for stating the following in the Funding Section of your manuscript:

“DM: grant IZSEZ_0183540 from the Swiss National Foundation for Science

DSC: project fund 100019_166010 from the Swiss National Foundation for Science

The funders had no role in study design, data collection and analysis, decision to publish, or preparation of the manuscript.”

We note that you have provided funding information that is not currently declared in your Funding Statement. However, funding information should not appear in the Acknowledgments section or other areas of your manuscript. We will only publish funding information present in the Funding Statement section of the online submission form.

Please remove any funding-related text from the manuscript and let us know how you would like to update your Funding Statement. Currently, your Funding Statement reads as follows:

“DM: grant IZSEZ_0183540 from the Swiss National Foundation for Science

DSC: project fund 100019_166010 from the Swiss National Foundation for Science

The funders had no role in study design, data collection and analysis, decision to publish, or preparation of the manuscript.”

Please include your amended statements within your cover letter; we will change the online submission form on your behalf

**The statement is correct. We removed funding information in the manuscript.**

4. Please include captions for your Supporting Information files at the end of your manuscript, and update any in-text citations to match accordingly. Please see our Supporting Information guidelines for more information: http://journals.plos.org/plosone/s/supporting-information.

**We added the caption for the supporting information at the end of the manuscript, as indicated in the guidelines**

# Reviewer #1:

Yoshinari UEHARA

R: In this study, the novelty of the study seems to be unclear. The authors should describe the novelty of this study a little more clearly.

**The authors would like to thank the reviewer for taking the time to review our study, and for this pertinent remark.
The novelty of our approach was indeed unclear. The main idea of our study was to change/improve Lewis and co-authors approach so that the calculation of the HRV decay rate can be performed based only on cardiac measurement. We thus intended to validate this approach using maximal effort test measurements, so that it can be used in the future with cardiac measurements yielded by portable devices (Holters, chest heart rate monitors).
The workload is not a valid option for such situation, for two main reasons:**

- **It is often not available for field measurements, such as for the 6 min walk test or for outside running cessions. The same holds for VO2.**
- **The approach using the workload as the independent variable of the exponential decay of HRV is only valid for non-null workloads. Indeed, during recovery, the HRV will have a huge variation during several minutes but would be associated with the same workload. This has been underlined in our manuscript in fig 3 and in the paragraph « comparison of models ».**

**We modified the introduction as follow, to clarify these points:
In the introduction, we added, starting at line 62:**

***Lewis and co-authors (11) modelled the decrease of the absolute high frequency (HF) and low frequency (LF) power of the RR series spectrum as an exponential decay of the workload. They find that the decay time (the HRV decay constant) of such exponential regression is highly correlated to the maximum work capacity of the athletes. The HRV decay constant constitute thus a promising index that could be used as a proxy of a person’ cardiorespiratory fitness (CRF). The CRF quantifies the ability to transport oxygen from atmosphere to the mitochondria, and is thus closely related to athletes’ performances, but is also a strong predictor of cardiovascular disease and all-cause mortality in the general population (20). The possibility to use the HRV decay constant to estimate the CRF using only cardiac data stemming from portable heart rate measurement devices, such as Holters or chest heart rate monitors, would be a major benefit. Indeed, CRF is usually measured either directly by the measured peak oxygen consumption, or derived from the maximum work rate achieved during a test, both methods requiring an important material setup. Estimating the CRF from cardiac measurements acquired during variable effort would extend the estimation of CRF to easier to implement effort test, such as the 6 min walk test, and to field measurements.***

***To do so, the approach of Lewis and co-authors has to be modified so that the HRV decay constant calculation does not rely on workload measurements, which cannot be measured with simple cardiac portable devices and are often not available from field measurements. Furthermore, the use of the workload as the independent variable in the calculation of the HRV decay constant is not valid during recovery periods. Indeed, during these periods, the HRV will increase back to its resting value and will be associated to the same null workload, thus invalidating the exponential relation between the workload and the HRV. We propose instead to analyze the decay of HRV during exercise as a function of the HR, because it is regulated during the exercise by the balance of the parasympathetic and sympathetic neuronal activity, which are both at the source of the HRV.***

***In the present study, we will therefore test the feasibility and the validity to calculate the HRV decay constant using the heart rate instead of the workload as the independent variable when modelling the exponential decay of HRV along exercise. This approach can be applied to resting, exercise and recovery periods, and only requires cardiac measurements as provided by portable devices.***

**In the discussion (line 303):**

***Our study aimed at validating the HRV decay constant approach developed by Lewis and co-authors using only cardiac measurements and a simple characterization of the HRV***

R: In addition, compared to previous studies that evaluated VT and LT, the method used in the previous study (using exercise intensity as the independent variable) seems to have better results than using heart rate as the independent variable in the present study from the perspective of evaluating exercise capacity.

**Concerning the apparent lower correlation found between HRV decay constant and MAP in our study when compared with the work of Lewis and co-authors, we realized that we did not proceed exactly as Lewis and co-authors. We modified our analysis, by using the HRV decay constant instead of the HRV decay rate (one is the inverse of the other, see new equation 2), and using linear Pearson correlation. We would like to stress the following points:**

- **In Lewis publication (11), when restricting their analysis per gender, they find a linear correlation of 0.58 - 0.65 (depending on the HRV index) between the HRV decay constant and the maximal workload. When doing the same (restricting the analysis on men or women, using the linear correlation coefficient and same HRV decay constant), we find similar values. We added this supplementary analysis to the result section, and we added to the discussion (line 310):

  *The linear correlation of 0.6 between the maximum aerobic power and the HRV decay constant we estimate is consistent with the results reported by Lewis and co-authors (12)***
- **In our study, we have a restricted population in term of performance and age when compared to the population studied by Lewis and co-authors, which could also restrict slightly the correlation between CRF and HRV decay constants (in the limitation section of the discussion (line 333):**

***The correlation between our HRV decay characterization and performance indices found among a heterogeneous population of athletes in term of sport modality is a strength. Indeed, although these different sport modalities require unequal sources of energy and train distinct physical capacities, leading to different cardiorespiratory and cardio autonomic control features, the exponential decay of HRV as a function of HR seems to be a robust indicator of the cardiovascular fitness*)**

R: Therefore, it is unclear why the authors used the research method with heart rate as the independent variable in the present study.

**We hope that the answers above clarified the interest of our initial choice.**

R: Furthermore, it is unclear why the authors used the measure of SDRR in this study, even though it is LF and HF in the frequency analysis in the previous study (citation #11). The authors should describe the reasons.

**There are various reason to choose SDRR in the present study, that we will list below:**

- **SDRR is the simplest measurement of HRV, whereas HF and LF powers require more complex mathematical modelling. It indeed requires to extrapolate the RR series to have equally time spaced points, it relies on the definition of the cutting frequencies. Following the aim to validate an approach easily implementable with cardiac-based measurements, we preferred to use the SDRR.**
- **Lewis and co-authors use the breathing frequency as the upper limit of the HF band. Following the aim as explained in our answer to your first point, our study aim at testing the HRV decay constant approach for cardiac based only measurements, for which we can consider that the breathing frequency is not measured.**
- **Lewis and co-authors, but also other authors, report contradictory results using spectral power HRV measurements, which from our point of view arise from the diversity of definitions and tools used. SDRR has the advantage of requiring little computation and of being easy to understand and implement.**

**We modified the text as follow, to further explain our choice (line 87):**

***We furthermore propose to use the standard deviation of the detrended RR intervals (SDRR) to estimate the HRV instead of spectral based calculation. Indeed, SDRR has been shown to be closely related to physical performances (22), is considered as a standard measure of HRV (4) and has the advantage of its simplicity, thus being easily implemented and reproduced. Frequency based measurements of HRV on the other hand may require to know the breathing frequency (11), which would not be available when using only cardiac measurements, and depend on the frequency limits used to define the low or high frequency bands of the spectrum (4) or on the norm used to express their values (8), thus leading to sometime contradictory results (23,24).***

R: The following three items are posted in the Introduction. The authors should provide details on these three items in the Discussion.

i) the inherent need to remove the main RR decrease due to the metabolic response to energy expenditure during exercise to obtain a detrended RR series, that is the beat-to-beat variability commonly designed as HRV;
ii) the technicity and the variety of the HRV characterizations, as illustrated by the variety of spectral measurements used and by the sometimes contradictory results they provide (20,21);
iii) the use of the mechanical work rate during exercise as the independent variable work rate during exercise as the independent variable of the exponential model, which could restrict the use of such approach only on RR series recorded during effort (HRV before and after). during effort (HRV before and after effort are associated to the same power value: 0 Watt) and may be protocol dependent."

**We removed this points from the introduction, as we rewrote this part of the introduction to answer the reviewer’ first points. We added the following sentence to the discussion to stress the advantage of our approach (line 315):**

***When compared to the approach studying the change of HRV as a function of the workload, our approach provides a better exponential variation of HRV and allows to consider period of measurements with a null workload but a strongly varying HRV, such as recovery periods.***

R: Methods; P 6/18; line 2
There is no mention of a rule regarding diet on the day of the study. The authors should describe any dietary stipulations, such as eating at least hours before exercise. Also, should provide detailed information on the exercise test, such as resting time before exercise and number of pedaling rate.

**All athletes were** **boarder of the CREPS and followed by a nutritionist from the CREPS boarding school. Effort tests were performed always at the same period of the day, thus the diet and eating time were similar between athletes of the study and the food intake was taken at least 2 hours before the exercise testing. Concerning information about the GET protocol, we complete the information in the methods section. The paragraph now reads (line 118-125):**

***The participants were instructed not to take alcohol, caffeine, nor to practice intense sport activities during the 24 hours preceding their GET. All athletes were boarder of the CREPS and were followed by a nutritionist, thus the diet and eating time were similar all the athletes of the study and the food intake was taken at least 2 hours before the exercise testing. The GET consisted of a 5 min of resting time before exercise, a 3 min cycling period at 50 watts, followed by a workload increase of 15 Watts every minute until exhaustion. Athletes were considered as exhausted when they were not able to maintain a pedaling rate over 60 rotation/min. At the end of the test, measurements were prolonged during a 3 min period to record the physiological recovery of the athletes. The participants were sitting during this recovery period.***

RESULTS;
R: It is unclear why the authors needed to include RECOVERY in the analysis. If you include it in the analysis, you should provide an explanation for this.

**As explained for the first point, the idea is to test the validity of a cardiac only based calculation of the HRV decay constant for different variable effort situation, including recovery. We hope the answer to your first point clarified why we included the recovery in the analysis.**

# Reviewer #2:

Thomas Gronwald

R: The present study „Decrease of heart rate variability during exercise: an index of cardiorespiratory fitness“ focuses on the effects exercise on HRV behavior as a potential marker of cardiorespiratory fitness.

I thank the authors for their effort and submission of the manuscript. All in all, the topic is very interesting and innovative having a non invasive method for HRV assessment and its practical application.

Unfortunately, there are no line numbers in the manuscript.

**We are deeply sorry that we forgot the line numbers. We added them in this new version.**

R: Abstract:
- Please use HRV as abbreviation after introduction

**Done**
R: - There are no results reported in the abstracts, can you provide any numbers and statistic outcomes?

**We added the following numeric results to the abstract (line 27-31):**

***The variation of this measure of HRV during exercise is properly fitted by an exponential decrease of the heart rate: the SDRR is divided by 2 every increase of heart rate of 20 beats/min. The HR increase necessary for the HRV to be divided by 2 is linearly inversely correlated with the maximum oxygen consumption (ρ=-0.60, p=0.006), the maximal aerobic power (ρ=-0.62, p=0.006), and, to a lesser extent, to the power at the ventilatory thresholds (ρ=-0.53, p=0.02 and ρ=-0.47, p=0.05 for the first and second threshold)***

R: Key Words:
- Some letters are capitalized

**Capitalized letters were removed**

R: Introduction:
- First sentence, reference is missing.

**We added a reference**

R: Methods:
- Please provide participant information at first, than study design, than anaylsis.

**We changed the order of the different subsections of the methods section.**

R: - First section, which effort is meant, which type of exercise

**We modified the sentence of the first section to clarify this point (line 101):**

***The database used in this work consists of records of a cycling graded effort test (GET) (Cf. the section below for details) performed by […]***

R: - Please provide reference for: Akaike information criterion (AIC) and Bayesian Information criterion (BIC).

**We added « Kuha J. AIC and BIC: Comparisons of Assumptions and Performance. Sociological Methods & Research. 2004 Nov 1;33(2):188–229. » as a reference for AIC and BIC**

R: - Please use VO2max consistently as abbrev.

**We tried to systematically use the VO2max abbreviation.**

R: - Do you use any ranges/threshold values for statistics analysis, eg Spearman coefficient

**For the interpretation of the correlation coefficient, we used the “Statistics at Square One” recommendation (**[**https://www.bmj.com/about-bmj/resources-readers/publications/statistics-square-one/11-correlation-and-regression**](https://www.bmj.com/about-bmj/resources-readers/publications/statistics-square-one/11-correlation-and-regression)**). We added the reference in the statistical analysis subsection of the methods section.**

R: Participants:
- First sentence: one bracket to much

**Corrected**

R: - In Table 1 you report very low VO2max values for young athletes, please check values

**We agreed with the reviewer that VO2max values appear to be low for athletes in national or regional divisions. Nonetheless it may be explained by the fact that 16 of the 18 athletes belonging to fencing or sprint kayak, two sports weakly associated to aerobic capacity. Furthermore, the 2 athletes practicing triathlon are in a regional division and are the youngest participants of the study (12 years old).**

**To be more transparent on this point we add the number of athletes in each discipline (line 104-105)**

**We checked the correlation between VO2max, and maximum workloads as well as power at ventilatory thresholds, and found highly significant linear correlations of 0.88, 0.73 and 0.85 respectively, indicating that the measures of VO2, even if shifted, present expected correlation with other aerobic measurements, thus ensuring the validity of our study.**

R: GET:
- Please use the word participants not subjects (in the whole manuscript)

**Done**

R: - Page 6: Participants were sitting during…

**The sentence reads now (line 125):**

***The participants were sitting during this recovery period.***

R: - …averaged other 5 breaths? „over“?

**Yes, thank you.**

R: Heart rate var.

- …in order them?
- …both being processes? (I recommend correction reading by a native speaker)

**We modified this two sentences, and thoroughly re-read the whole manuscript.**

R: Statistical analysis
- Some repitition to earlier provided information, please rewrite

**We removed mention of AIC and BIC before the section statistical analysis, to avoid repetitions**

R: Results
- Correlation interpretation in methods section is missing, r values of 0.5/0.6 is at a moderate level.

**Following Swinscow’s « Statistics at square one » textbook : « If we wish to label the strength of the association, for absolute values of r, 0-0.19 is regarded as very weak, 0.2-0.39 as weak, 0.40-0.59 as moderate, 0.6-0.79 as strong and 0.8-1 as very strong correlation ».**

**As explained in the introductory paragraph of the rebuttal letter, we recalculated the correlation but using the decay time constant as defined by Lewis and co-authors (the amount of HR increase that divide the SDRR per 2) and the Pearson linear correlation.**

**The description of the correlations now reads (line 280):**

***The HRV decay constant*** $\boldsymbol{\tau}_{\boldsymbol{HR}}$ ***of SDRR as a function of HR is strongly and significantly inversely correlated with the maximum power reached during effort test MAP (ρ = -0.62, p = 0.006) and with VO2max (ρ = -0.60, p = 0.009), and moderately inversely correlated with the power at the ventilatory thresholds (ρ = - 0.53, p = 0.02 and -0.47, p = 0.05 for PVT1 and PVT2).***

R: Discussion
- First section: „strongly related“ there are only corr. coeff. values of around 0.5/0.6; please revise

**We removed the word strong from the discussion**

R: - „This HRV decay rate increases (i.e. a faster decay of HRV when HR increases) for athletes with higher aerobic capacity.“ Where do you get this result, there is no subsection for different performance level.

**This interpretation stems from the fact that we find a linear correlation between the HRV decay constant and VO2max or maximum aerobic power (which are measurements of aerobic capacities of the athletes). This means that the decay rate is higher for athletes with higher aerobic capacities (or the decay constant smaller)**

R: - „The strong correlation between our HRV decay characterization and performance indices found among a heterogeneous population of athletes in term of sport modality is a strength.“ Please revise strong correlation (see above)

**Done**

R: - All in all, what do the results mean for the individual athlete in ist application (last sentence of conclusion: „ave the way to the measurement of cardiorespiratory fitness using measurements provided by mobile devices“)? On group level you have moderate correlations with performance markers, that given, I would say its very unclear whether your model fits for the individual athlete.

**Though we understand the reviewer’s point of view, the results we obtain are similar to the ones from Lewis and co-authors. In the section “Study of individual decrease of HRV during effort”, the authors calculated the HRV decay constant for each athletes’ effort test, and found a linear correlation of 0.6 between the HRV decay constant and the peak power achieved during the test and with the VO2max.**

**We would like to stress out that heart rate recovery, which is extensively used in medicine (as a predictor of mortality or cardiovascular adverse events) and in sports sience, has been found to have similar moderate correlation (or sometimes lower) with VO2max or performance markers for young athletes (see for example** [**http://dx.doi.org/10.1007/s00421-011-2014-0**](http://dx.doi.org/10.1007/s00421-011-2014-0) **) and for cardiac patients (**[**http://dx.doi.org/10.1016/j.healun.2006.04.006**](http://dx.doi.org/10.1016/j.healun.2006.04.006) **). But HRR enters several VO2max prediction equations that are used/have been used at the individual level (some examples:** [**http://dx.doi.org/10.1136/jrnms-74-107**](http://dx.doi.org/10.1136/jrnms-74-107) **or** [**http://dx.doi.org/10.1080/00140137708931616**](http://dx.doi.org/10.1080/00140137708931616) **for the oldest versions used for step tests,** [**http://dx.doi.org/10.1093/ptj/70.11.734**](http://dx.doi.org/10.1093/ptj/70.11.734) **for a more recent version), and it has been shown to be a valid index to follow training changes of athletes (see** [**http://dx.doi.org/10.1007/s40279-016-0484-2**](http://dx.doi.org/10.1007/s40279-016-0484-2) **). In medicine, although the correlation is only moderate with VO2max, the use of a threshold on heart rate recovery has been proven to be a good and easy to implement predictor of CAD and deaths (see** [**http://dx.doi.org/10.7326/0003-4819-132-7-200004040-00007**](http://dx.doi.org/10.7326/0003-4819-132-7-200004040-00007) **,** [**http://dx.doi.org/10.1056/NEJMoa043012**](http://dx.doi.org/10.1056/NEJMoa043012) **or** [**http://dx.doi.org/10.1016/S0735-1097(01)01652-7**](http://dx.doi.org/10.1016/S0735-1097(01)01652-7) **), which is widely used in medical practice.**

**We agree that as is, our analysis cannot be used for the prediction of VO2max at the individual level. Nevertheless, we think that similarly to what has been done with heart rate recovery, our analysis can be used as an index of CRF to compare groups in scientific studies, that changes of HRV decay constant could be used to follow CRF changes, that future studies could find thresholds values of the HRV decay constant predicting CAD and thus in daily practise in medicine, and that this index could be part of more complex equation (to be developed) able to predict individual VO2max.**

**We added to the limitation subsection of the conclusion (line 344):**

***The rather moderate correlation found between HRV decay constant and CRF indices is not high enough to use our analysis to predict CRF at the individual level. Nevertheless, this correlation is similar to those obtained between heart rate recovery and VO2max or maximal workload (44–46). Therefore, the HRV decay rate could find applications similar to those of heart rate recovery, such as the use of threshold values to detect cardiovascular problems or risk of deaths (47–49), the monitoring of CRF or training changes (41) , or being part of more complex equation with higher predictability (50,51).***

R: - Please provide an extra section for limitations.

**Done**

# Reviewer #3:

Giovanna Zimatore

R: This study investigates cardiac beat-by-beat measurements during maximal graded effort test; it can support other methods to consider the post-processing Heart Rate detections in promising tools for the study of cardiac measurements generated by portable devices with the aim of obtaining best performance in athletes. HRV was studied in function of the corresponding HR and it was confirmed that SDRR behaves as an exponential of the heart rate.
It seems very interesting, however, some points need to be better clarified. I think it is necessary, for example, in the introduction that a brief paragraph describing what CFR indices are and why it would be important to detect them.

**We thank the reviewer for helping us improving the manuscript. We added in the introduction:**

***The HRV decay constant constitute thus a promising index that could be used as a proxy of a person’ cardiorespiratory fitness (CRF). The CRF quantifies the ability to transport oxygen from atmosphere to the mitochondria, and is thus closely related to athletes’ performances, but is also a strong predictor of cardiovascular disease and all-cause mortality in the general population (20).***

R: The Akaike information criterion (AIC) and Bayesian Information criterion (BIC) can be briefly described in method or in appendix).

**We added the following reference for the AIC and BIC**

**Kuha J. AIC and BIC: Comparisons of Assumptions and Performance. Sociological Methods & Research. 2004 Nov 1;33(2):188–229.**

R: However, the main question is about the model that would describe the evolution of SDRR along the exercise (in the whole exercise duration time) by an exponential equation (in three different forms) in function of mean HR, power and workload. The parameter a and b in the equations are found by sliding windows along the exercise.

**The model we use is based on an outcome which is indeed calculated in successive windows (and not sliding windows), resulting in a median of 22 [19, 28] SDRR and median HR measurements per effort tests. The exponential models are then applied to these series of measurements.**

**We added, in the anaylsis section subsection of the methods section (line 137):**

***HRV during each individual effort was then quantified by the SDRR, calculated on adjacent windows of one minute (corresponding to each effort step during effort, see “Heart rate variability” subsection). The mean HR, the mean work intensity %VO2max and the mean work load were calculated in the same windows. The resulting series of SDRR values was then analyzed in two different parts.***

**We added the following paragraph to the result section:**

***The calculation of SDRR, mean HR, mean power or mean work intensity in adjacent windows of 1 minutes for each detrended RR series yielded for each effort test a series of measurement with a median number of 22 [19, 28] points.***

R: Table 3 and Fig4 are refer only to model 1 (it may be better to change the title of the paragraph in “Study of individual decrease of HRV during effort in model 1”), this result must be emphasized.

**In order to clarify this, we decided to name differently the coefficient of the 3 models in equation 2, to avoid confusion between them. We also decided to consider the HRV decay constant, as proposed by Lewis and co-authors in (11) instead of the HRV decay rate:**

$\begin{matrix} SDRR_{j}=b_{HR}e^{\frac{-\ln\left( 2 \right)\times HR_{j}}{\tau_{HR}}} (model 1) \\ SDRR_{j}=b_{P}e^{\frac{-\ln\left( 2 \right)\times P_{j}}{\tau_{P}}} (model 2) \\ SDRR_{j}=b_{I}e^{\frac{-\ln\left( 2 \right)\times I_{j}}{\tau_{I}}} (model 3) \end{matrix}$

**We referred to the exponential decays in Table 3 and fig 4 as** $\boldsymbol{\tau}_{\boldsymbol{HR}}$ **and** $\boldsymbol{b}_{\boldsymbol{HR}}$**, and added model 1 to the subsection title, as suggested by the reviewer.**

R: The last part it is not clear in my opinion. How can Stationarity hypothesis in HR be assessed in window shorter than 30 sec (or 50 points)?

**When using the polynomial detrending, we actually substract to each point** $\boldsymbol{x}_{\boldsymbol{i}}$ **the polynomial trend estimated within a windows centered on** $\boldsymbol{x}_{\boldsymbol{i}}$**. The 0^th^ order polynomial detrending correspond to substracting the moving average of the RR series. The first order polynomial detrending is substracting the moving estimate of the local linear trend**

R: The fig.3 it is impressive, but it seems not to be necessary, or the significance must be better explained.

**The authors have to respectfully disagree with the reviewer: The Fig 3 describes the three representations of the 3 models of equation 2 and their estimation on the ensemble of all the SDRR values. The entire paragraph of the “comparison of models” subsection of the results refers to this figure.
It could be that the reviewer refers to figure 4. In this case, to answer the reviewer’s last two comments, the figure have been changed and simplified, and we believe is now easier to understand.**

Specific comments

R: Abstract
Please rewrite the sentence: “It indicates that among athletes with better fitness, HRV has higher values at low heart rate and decreases faster when the heart rate increases during exercise”.
This is a key point. HRV has higher values at low heart rate it is well-known, it must be emphasized that HRV “decrease faster”

**We agree with the reviewer, and kept only the part about the decrease of the HRV. The sentence now reads:**

***It indicates that among athletes with better fitness, HRV decreases faster when the heart rate increases during exercise.***

**We furthermore rewrote the result section to insist more on this point:**

***The HRV decay constant*** $\boldsymbol{\tau}_{\boldsymbol{HR}}$ ***of SDRR as a function of HR is strongly and significantly inversely correlated with the maximum power reached during effort test MAP (ρ = -0.62, p = 0.006) and with VO2max (ρ = -0.60, p = 0.009), and moderately inversely correlated with the power at the ventilatory thresholds (ρ = - 0.53, p = 0.02 and -0.47, p = 0.05 for PVT1 and PVT2). The fact that the HRV constant, i.e. the HR increase it takes to divide SDRR by two, decrease when the CRF indices increase means that the decrease of SDRR with HR is faster among athletes with better CRF.***

R: Introduction
How can measure the decrease of their variability in frequency domain?

**It is the variability that is measured in the frequency domain (HRV can be measured by several quantification of the RR series spectrum), not its decrease. We modified the sentence as follow:**

***decrease of their variability measured by both time and frequency HRV indices***

R: Pag. 3
“such as the decrease of their variability measured both in time and frequency domain (8,10–15), the modification of the scaling properties of their linear correlations (16), or even the reduction of their sample entropy (17) and of their nonlinear correlations (18,19)”.
Too more information in two and half rows, please explain more in details and add even the more recent paper of [19] in PlosOne.

**The authors cited the more recent plosOne paper for reference 19. We extended the paragraph, which now reads:**

***The progressive withdrawal of the parasympathetic activity and the subsequent increase of the sympathetic activity causes extensive changes in RR intervals. For example, it is well stablished that the variability of RR intervals (HRV) decreases both in time and frequency according to several HRV indexes, including coarse graining spectral analysis (10), Poincaré plots (11), HRV spectral power in different frequency bands (12–14), or statistical properties of the increments of RR intervals (15,16). But the decrease of HRV is not the only effect of exercise: it is also known that exercise modifies the linear correlations of the RR intervals as measured by Detrended Fluctuation Analysis (17), and produces a reduction of their sample entropy (18), pointing to a reduction of the complexity of the cardiac signal associated to exercise. Similar conclusions can be drawn by measuring the nonlinear correlations of RR intervals, which are also known to decrease with exercise (19,20).***

R: Pag 3 last row:
Add a definition and description cardiorespiratory fitness (CRF) indexes that are only mentioned at pag 4 :CRF indices (namely maximum VO2, maximum aerobic power, power at ventilator thresholds).

**These indices are described already in the “Graded effort test (GET) measurement” subsection:**

***The ventilatory thresholds 1 (VT1) and 2 (VT2) were calculated using the Wasserman method (24). Maximum aerobic power is the maximum power achieved during the last completed step of the GET. Peak HR and VO2max are the maximum values of the HR and VO2 averaged over 5 breaths.***

R: Pag 3 Row 15 replace work load with workload

**Done**
R: Pag 3 i) Replace designed with defined

**We removed this paragraph to answer reviewer1’s remarks**

R:Please explain stationarity hypothesis in HR

**We do not make the hypotheses that HR is stationary. It is actually not during the effort test. Therefore, we subtract the global HR trend during the effort by fitting its global evolution along effort using a validated dynamical analysis (a first order differential equation). The high R2 of the regression ensure us that we substract the long term changes of HR.**

**In order to avoid confusion, we removed the word stationary, and added to the introduction:**

***To distinguish the HRV from the global change of the RR intervals due to the increase of HR caused by the workload changes, we will estimate the change of RR along the effort test using a recent validated dynamical model (21) to obtain a detrended RR series.***

**In the methods section, in analysis, we modified the first sentence as follow:**

***Each individual RR series was first detrended to remove the global RR changes due to the heart rate adaptation to the workload changes.***

R: Methods
Please, provide more information about sample.
For example: age, anthropometric measurements,

**The first sentence of the methods section now reads, line 97:**

***The database used in this work consists of records of a cycling graded effort test (GET) (Cf. the section below for details) performed by 18 young athletes (10 males and 8 females; 15.2 [14.0, 16.8] year-old, 174.0 cm [165.0, 182.0] height and 62.9 kg [54.3, 76.5] weight, see table 1) of the Regional Physical and Sports Education Centre (CREPS) of French West Indies (Guadeloupe, France),[…]***

R: explain why you can consider male and female together despite the well-known gender differences existing in physical exercises calculated on adjacent windows of one minute (corresponding to each effort step during effort, see “Heart rate variability” subsection).

**There actually is no report of a gender/sex difference on the rate of decrease of HRV during exercise in the literature. Lewis and co-authors analysed both male and female together, and ran a separate analysis as a complement. A second argument to consider both male and females together is to have a higher power to detect the correlation, and to span a higher range of VO2max and peak workload values.
We performed a separate analysis per sex, and added in the result section:**

***Performing the same analysis but separately on males and females yielded higher linear correlation coefficients, but only significant for the correlation between*** $\boldsymbol{\tau}_{\boldsymbol{HR}}$ ***and VO2max (ρ=-0.68, p = 0.01) and MAP (ρ=-0.66, p = 0.02) for males.***

R: It is not clear and easy-readable: Please rewrite, describe the timing of protocol clearly, simply explain that the workload changes every minute. (see pag7)

**According to the reviewer comment we changed the sentence describing the GET protocol to facilitate its understanding (line 117):**

***The GET consisted of a 5 min of resting time before exercise, a 3 min cycling period at 50 watts, followed by a workload increase of 15 Watts every minute until exhaustion. Athletes were considered as exhausted when they were not able to maintain a pedaling rate over 60 rotation/min. At the end of the test, measurements were prolonged during a 3 min period to record the physiological recovery of the athletes. The participants were sitting during this recovery period.***

R: Pag 7 “Detrending”: what are the differences among 23-24-25?

**23 is the reference describing the simulation study yielding the validity of the statistical approach to estimate the coefficient of a first differential equation with multiple excitations; 24 and 25 are two validation studies of its use for heart rate data during effort tests.**

R: AIC and BIC can be briefly descried in method or in appendix

**We added a reference and the following text:**

***All signal processing and statistical analysis have been performed with the R 4.1 open source software (29). The comparison between regression models is based on Akaike information criterion (AIC) and Bayesian Information criterion (BIC) (30), which are two estimator of the prediction error used to compare regression models.***

R: Pag 7 last rows
Because HRV is a result of parasympathetic and sympathetic neuronal activity, both being processes taking part in the regulation of the mean HR, we propose to analyze the decay of SDRR during exercise as a function of the mean HR calculated on the same time windows.

It is not clear because the second part (“we propose to analyze …” is a consequence of the first part “Because HRV is …)

**We moved this sentence to the introduction, and rephrased it as follow:**

***We propose instead to analyze the decay of HRV during exercise as a function of the HR, because the latter is regulated during the exercise by the balance of the parasympathetic and sympathetic neuronal activity, which are both at the source of the HRV.***

R: Results
Pag 9 Explain better the first three rows
“The median number of RR distance recorded was 2726 beats (Inter Quartile Range [2433;3548]). An example of detrended RR estimated using the dynamical model presented in the method section is illustrated in Fig 2. This model produced an individual estimation of RR with a median R2 of 0.97 [IQR: 0.91 – 0.98].

**We rewrote this paragraph as follows:**

***The median number of RR distance recorded during the GETs was 2726 beats (Inter Quartile Range [2433; 3548]). The dynamical model based on first order differential equation was used to estimate the global changes of RR along each GET, and yielded individual estimations of RR with a median R2 of 0.97 [IQR: 0.91 – 0.98]. An example of detrended RR estimated using this dynamical model is illustrated in Fig 2. The calculation of SDRR, mean HR, mean power or mean work intensity in adjacent windows of 1 minutes for each detrended RR series yielded for series of measurement with a median number of 22 [19, 28] points.***

R: Pag. 10 add to the first sentence something like: “for these reason in the following ……only model 1…”

**We added:**

***For these reasons, we will use this model for the rest of our analyses***

R: In Fig3 Please add three panels with only “test”

**As we understand the reviewer’s remark, she would like to better see the measurement during the non-nul workload. In order to avoid too complex figure, we decided to colour the SDRR measurements depending on the GET stage, i.e if they corresponded to pre-effort, during the effort, or post effort (recovery), so that the different stages of the GET are more visible. We furthermore displayed the estimated result of the regression models performed on the “test” only. The figures is now:**

**We hope these changes answer the reviewer’s expectations.**

R: In Caption of fig3 A comma is missing before “respectively”: in A, B and C, respectively

**Done**

R: Pag 12 table 3 please correct “correlation”

**corrected**

R: (HRV decay rate a and the HRV at HR=0 b) change “a, HRV decay rate and b, HRV decay rate at HR=0 “

**Done**

R: Can you better explain the difference between ωRR and ωp ?
Please explain because it would be trivial to consider ωRR and ωp with the same number of points
In my opinion it is not clear to catch the differences between ωRR and ωp

$\boldsymbol{\omega}_{\boldsymbol{p}}$ **had to do with the polynomial detrending used in the sensitivity analysis: it was the size of the rolling windows used to detrend the RR series (for each point, a polynomial trend is estimated within the windows of size** $\boldsymbol{\omega}_{\boldsymbol{p}}$ **centered on each point and subtracted).**

$\boldsymbol{\omega}_{\boldsymbol{RR}}$ **was the size of the adjacent windows used to calculate the SDRR on the detrended RR series.**

**There was no apparent reason to choose the same value for both, thus our initial decision to study the influence of these two parameters. But we understand the reviewer’s point, and agree that the figure 4 is also unnecessary complicated. We thus propose to set both windows size to the same value** $\boldsymbol{\omega}$**, and study the influence of this single parameter in the sensitivity study. We changed along manuscript, and modified the figure 4. The figure is now:**

***Fig 4. Pearson correlation coefficient and associated p value (p < 0.05 or p >= 0.05) found between HRV decay constant and VO2max as a function of the windows size*** $\boldsymbol{\omega}$ ***when using a 0^th^ (p=0) order polynomial detrending.***

**The supplementary figure, presenting the sensitivity analysis for all CRF indices and the 3 polynomial order has been updated accordingly.
We also added to the “sensitivity analysis” subsection of the method section:**

***The polynomial detrending procedure consist in subtracting to each point of the RR series the estimated polynomial trend estimated within a centered windows of*** $\boldsymbol{\omega}$ ***points.
In more detail, it can be described as follows: let us consider a time series of RR intervals […]***

R: Pag 13 athletes with higher aerobic capacity….. Add references

**We changed “aerobic capacity” with “athletes with higher cardiorespiratory fitness”. This stems directly from our results.**

R: In the fig3, to what correspond the horizontal line below 50 appears in all the panels?

**The horizontal line is a numerical artefact: when using a windows size of 41 points to calculate the SDRR, the values of the HRV decay constant do not significantly correlate anymore with VO2max, whatever the windows size of the detrending used. This artefact does not appear anymore, as the figure has been changed to answer your previous point.**
